# Interventions that prolong multidimensional healthspan in humans: a systematic review of randomized controlled trials

**DOI:** 10.1093/gerona/glag133

**Published:** 2026-05-22

**Authors:** Haoxin Tina Zheng, Aung Zaw Zaw Phyo, Claire McCubbin, Zimu Wu, Heike A Bischoff-Ferrari, Joanne Ryan

**Affiliations:** School of Public Health and Preventive Medicine, Monash University, Melbourne, Victoria, Australia; School of Public Health and Preventive Medicine, Monash University, Melbourne, Victoria, Australia; School of Public Health and Preventive Medicine, Monash University, Melbourne, Victoria, Australia; School of Public Health and Preventive Medicine, Monash University, Melbourne, Victoria, Australia; Department of Acute Aging Medicine Felix Platter, University of Basel, Basel, Switzerland; School of Public Health and Preventive Medicine, Monash University, Melbourne, Victoria, Australia; (Medical Sciences Section)

**Keywords:** Healthspan, Healthy aging, Intrinsic capacity, Quality of life, Randomized controlled trials

## Abstract

Maximizing healthspan, the period of life spent in good health, is a public health priority. This review aimed to summarize the current evidence from randomized controlled trials on interventions that can prolong healthspan in humans. The specific focus was on multidimensional person-centered outcomes, which reflect functioning rather than disease, such as intrinsic capacity and quality of life. This review was registered (CRD420251015780) and conducted with adherence to the PRISMA guidelines. MEDLINE, EMBASE, and grey literature were systematically searched for studies evaluating interventions that improve intrinsic capacity or quality of life. The search included articles published up to October 16, 2025. Of 1960 publications identified, 15 articles (4656 participants) met the inclusion criteria. There was high heterogeneity between the included studies in terms of the interventions examined, which varied from exercise alone (7 studies) to multidomain interventions (6 studies, all of which included an exercise component), daily oral supplementation (2 studies), or caloric restriction (1 study). Overall, 11 studies reported that exercise or multidimensional intervention (which included an exercise component) improved intrinsic capacity and quality of life. However, due to the small number of studies and heterogeneity, no conclusion could be drawn regarding other interventions. Therefore there is some evidence that exercise could extend intrinsic capacity and quality of life, either aerobic or resistance training alone, or a combination of different types of exercise; but further research is required to evaluate the effect of other interventions that may prolong healthspan across more diverse populations.

## Introduction

Rapid advancements in treatment, prevention, and awareness of chronic diseases have significantly extended human lifespan compared to previous decades.^1^^,^^2^ This increase in lifespan has led to remarkable growth in the aging population.^3^ This demographic shift places a considerable burden on healthcare systems, as increased lifespan does not always equate to more years lived in good health. Indeed, with aging occurs a general deterioration in bodily systems, including a loss of physiological reserve and chronic low-grade inflammation. Many diseases become more common with aging,^4^ and on average, an individual is expected to live with disability for 9 years before their death.^1^ This can negatively impact an individual’s quality of life and overall well-being.^5^^,^^6^ Determining ways to prolong healthy lifespan is thus an important priority.^7^^,^^8^

Healthy life expectancy, or healthspan, is the period of life spent in good health.^8^^,^^9^ There is no single definition of healthspan, with a recent review reporting more than 100 definitions that have been used in the literature.^10^ Intrinsic capacity (IC), defined as the sum of an individual’s physical and mental abilities, has been proposed as an integrated measure of healthspan.^11^ Rather than a disease lens, IC focuses on the capacity of individuals to maintain good health and general well-being with aging. Similarly, quality of life (QoL) is a well-established multidimensional indicator that reflects an individual’s overall well-being and personal perception of life,^12^ offering a comprehensive but more subjective measure of health. While these two measures differ from other definitions, including those that focus on multimorbidity, the commonality is a person-centered focus on functioning rather than diagnoses. This aligns with a more holistic geroscience approach, which posits that targeting the underlying mechanisms of aging^4^^,^^8^^,^^9^^,^^13–15^ may be most beneficial for extending healthspan.^4^^,^^15^

Studies using animal models have provided quite compelling evidence to support a link between lifestyle and behavioral interventions and indicators of healthspan (namely, enhanced physical capacity).^16–19^ Other studies have shown that pharmacological interventions and supplements are effective in preventing the decline in health status in mammalian models.^20^ Metformin, rapamycin, and resveratrol are the most studied drugs for their promising effects in restoring metabolic functions.^21–27^

In humans, there have been a small number of studies that have specifically focused on composite measures of healthspan as outcomes. However, unlike studies of disease risk or longevity, most of this research has looked at healthy lifestyle behaviors as the most effective approach to preserve function and delay the onset of disease.^14^ For example, physical activity has been widely investigated for its beneficial role in decreasing the risk of age-related diseases and increasing lifespan.^28^ Dietary interventions, especially caloric restriction (CR), which involves reducing the intake of energy, have been associated with longer lifespan and better health outcomes.^2^^,^^16^^,^^29–32^ Stress management and wellness programs have also been shown to improve the levels of biomarkers that are associated with disease risk.^33^^,^^34^ Pharmacological supplementation may also be effective.^16^^,^^20^ Liao et al. indicated that β-nicotinamide mononucleotide (NMN) could enhance aerobic capacity.^35^ Additionally, combining exercise, diet, and pharmacological treatments may be beneficial in extending healthspan.^20^

There have been a couple of systematic reviews investigating the effect of multidomain interventions on individual components of (e.g., locomotion or cognition), but not overall IC.^36^^,^^37^ This systematic review aimed to synthesize the existing evidence in humans for interventions that could be effective in prolonging multidimensional indicators of functional well-being with age, including IC, a multi-component measure of health, and QoL, an indicator of the self-perception of overall health. All types of interventions were considered, including single-component and multidomain.

## Material and methods

This systematic review was registered with the International Prospective Register of Ongoing Systematic Reviews (PROSPERO),^38^ with the registration number CRD420251015780, and conducted in accordance with the Preferred Reporting Items for Systematic Reviews and Meta-analyses (PRISMA) guidelines.^39^

### Literature sources and search strategy

A systematic search of MEDLINE (Ovid) and EMBASE via Ovid was conducted using predefined search terms to identify studies examining any intervention and its effect on multidimensional measures of healthspan, including IC or QoL, as an outcome. To avoid missing any possible studies that examined interventions that were not previously described, no specific interventions were searched. Ongoing trials in the clinical trials register that is maintained by the US National Institute of Health (http://clinicaltrials.gov) were used as a supplementary search. Reference lists of the included trials and other relevant review articles were retrieved to identify any other relevant articles. Eligible articles were those published in English until 16 October, 2025. The complete list of search terms and strategy is available in the Supplementary Material.

### Selection criteria

#### Types of studies

Randomized controlled trials (RCTs) of any design, including cross-over, parallel, and cluster, that investigated any type of intervention and the effect on healthspan were eligible for inclusion. Publications that were not available in full text, such as conference abstracts, were ineligible.

#### Types of participants

Any trial that enrolled participants aged over 18 years was eligible. To maximize inclusivity, no age restriction was applied. Studies involving animal models (in vitro or in vivo) were excluded.

#### Types of interventions and comparators

Included were trials that evaluated any behavioral, psychological, or pharmacological intervention, including diet, exercise, treatments/supplements, medication, and others. This included single (e.g., intervention with exercise only) and multidomain interventions, and no interventions were excluded. Comparators included any group that did not receive the specific intervention, including placebos or alternative active comparators or doses.

#### Types of outcome measures

Studies were included if they investigated the effect of the intervention on healthspan measured by multidimensional indices of function, such as IC and QoL. There are multiple definitions of healthspan, which can refer to biological aging, biological measures, or phenotypic outcomes. Multimorbidity is one of the common definitions that is used, but it is a disease-centered perspective.^10^ For this review, we included all studies that were focused on healthy longevity, healthy aging, or healthspan (in the title, abstract, or body of the text), and defined their outcome as an overall multifaceted measure of function. This, therefore, included IC, a multidimensional measure of functional ability^11^ that includes locomotion, vitality, cognition, psychological, and sensory components.^11^^,^^40^ QoL, which captures multiple perspectives of well-being at the individual level and self-perception, was also eligible for inclusion. Other multicomponent indicators that capture more than one aspect of physical and psychological functioning were eligible as secondary outcomes of this review. Studies that focused purely on biomarkers associated with health status, longevity, genetic variants associated with longevity, or physical or cognitive performance individually, or only measures of disease were excluded.

#### The timing of outcome assessment

Outcomes measured at any time point were considered, with no minimum or maximum period.

### Selection of studies

All eligible studies were retrieved and deduplicated using EndNote X9 (Clarivate, Philadelphia). Article screening and selection were conducted using Covidence (Covidence, Melbourne, Australia). Two researchers (HTZ, AZZP) independently screened titles and abstracts, and all conflicts were resolved through discussion or consultation with a third reviewer. Full text screening of the retained studies was conducted for final eligibility.

### Data extraction and management

Three reviewers (HTZ, AZZP, CM) independently extracted data using a data extraction form developed for this review. The reviewers resolved any discrepancies through discussion with a fourth reviewer (JR). The information extracted included: first author, year, country, study design, number of participants, mean age of participants, sex, ethnicity, description of the intervention and comparison group(s), outcome measure(s) and timing of assessment, as well as the summary findings (if the outcome was assessed at multiple time points, results were extracted that related to the main time point that was prespecified, or if no main time point was indicated, the end of the intervention and/or multiple time points were presented).

### Risk of bias assessment

Risk of bias (RoB) was assessed using the Risk of Bias Tool (RoB 2) for randomized controlled trials.^41^ Two researchers (HTZ, CM) individually assessed the RoB of each study, with a third researcher involved in the case of conflicts. Judgements on RoB for each domain were presented in Table S1.

### Data synthesis and presentation of results

The results are presented qualitatively and grouped according to similar interventions. No meta-analysis was conducted because of the large heterogeneity across studies in terms of both the interventions examined and the exact healthspan outcome that was assessed.

## Results

### Search results

**Figure 1:**
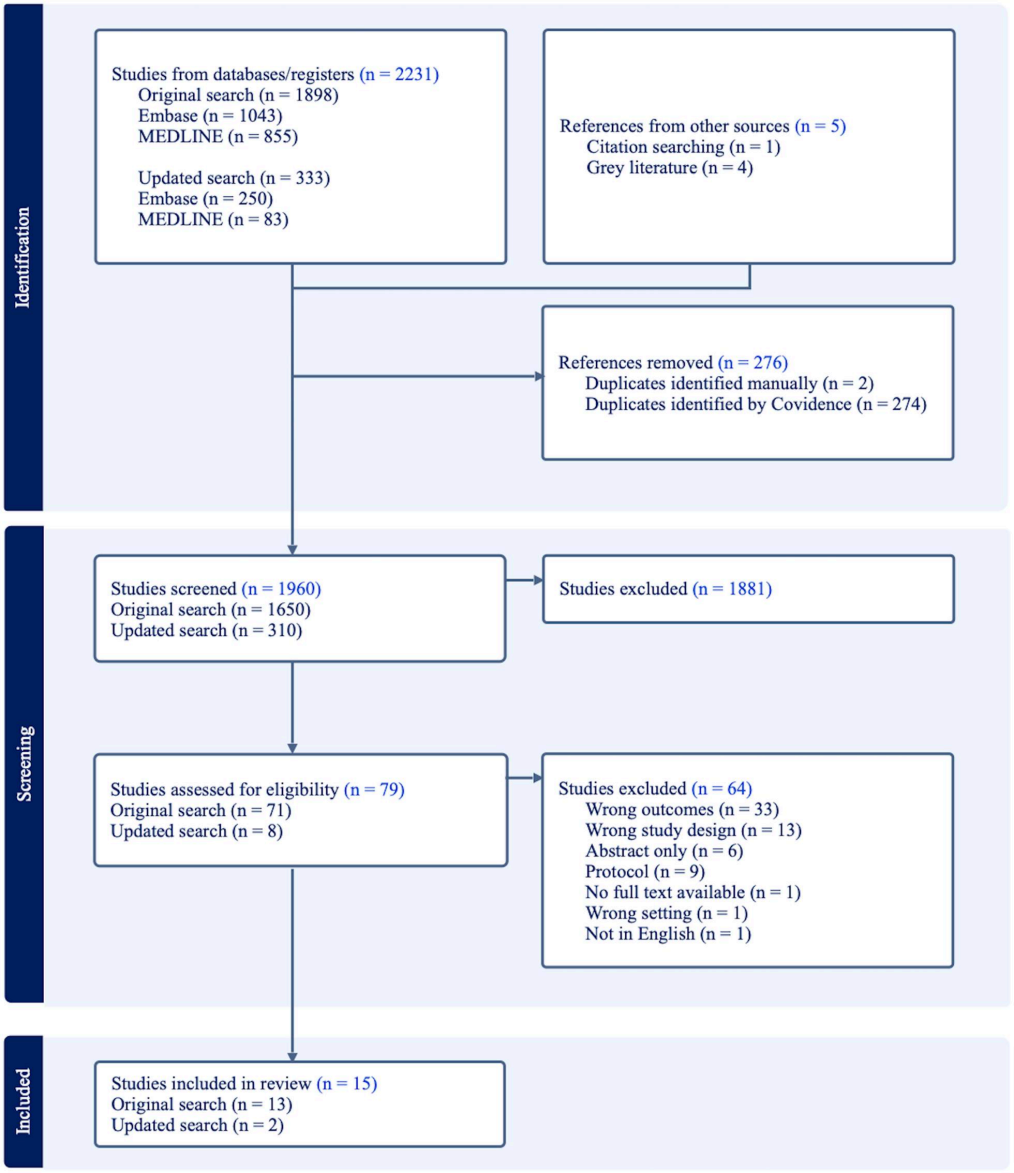
Flow diagram for study selection. The original search covered publications up to March 21, 2025, and the updated search included articles published between March 22, 2025 and October 16, 2025.

The search identified 1960 citations after removal of duplicates. Seventy-one studies were selected for full-text review, and 56 of these were excluded for the following reasons: wrong outcomes (33 studies), wrong study design (13 studies), abstract only (6 studies), a study protocol rather than original research (9 studies), not in English (1 study), and not available in full text (1 study, noting that several attempts were made to retrieve the full text, including contacting the authors directly). After screening, 15 articles were eligible for inclusion in this review, and all publications reported on unique studies. A flow diagram for study selection, using the PRISMA template (available at: http://www.prisma-statement.org/), is presented in Figure 1.

### Description of included studies

The studies included^42–56^ were conducted in Europe (6 studies), the USA (3 studies), and Asia (6 studies). Characteristics of included studies are presented in Table 1.

**Table 1:** Characteristics of the studies included in this systematic review by intervention type.

Author	Study design	No. of participants (intervention), population (country)	Mean age (*SD*), sex (% female), ethnicity (%)	Intervention (description)	Measure of healthspan	Summary findings, mean estimate (*SD*)/[*SE*], effect estimates [95% CI], and *p* value
**Multidomain interventions**						
**Giudici et al.^48^**	RCT	1445 (356), community-dwelling older adults without dementia (France)	75.3 (4.4), 64.2 ♀, NM	12-month multidomain intervention (group sessions of cognitive stimulation, physical activity, and nutritional counseling (group session) +/− Omega-3 PUFA (ω-3 supplementary)	IC *z*-score: the mean *z*-score of 4 domains： cognition, locomotion, psychological, and vitality	No effect for multidomain +ω-3: β = 0.04 [−0.05, 0.12] *p* = .7844 No effect for multidomain alone: *β* = 0.07 [−0.02, 0.16] *p* = .363 No effect for ω-3 alone: *β* = 0.01 [−0.08, 0.10] *p* = .835
**Chi et al.^47^**	RCT	195 (100), Community-based elderly aged ≥65 (China)	Intervention: age: 65-69 57.9; 70-74 26.3; ≥75 15.8, 76.8 ♀, NM Control: Age: 65-69 67; 70-74 31; ≥75 2, 64.0 ♀, NM	5-week Community Aged Well-being Class (CAWC) included physical activity, emotional health, and cognitive function training, and social participation with 19 weeks of follow-up	Self-Rated Active Aging Scale: 15 items covering self-daily life management, cognition of aging process, and self-esteem, physical and psychological maintenance, family and friend relationships, economic and environmental safety	Intervention + effect: Follow-up 1 (7 weeks): *β* = 13.91 [0.44] *p* < .001; End of study (19 weeks): *β* =11.48 [0.91] *p* < .001
**Yaffe et al.^50^**	RCT	172 (82), individuals at high risk of dementia with ≥2 risk factors (USA)	Intervention: 75.8 (4.9), 69.5 ♀, 78 White Control: 75.6 (4.7), 56.7 ♀, 84.4 White	2-year personalized activities set by the health coach, which included physical activity and brain training	1. QoL PROMIS Global Health measure Composite *z*-score: based on physical activity, blood pressure, sleep quality, use of medications, mental health, smoking status, and hemoglobin A1c value	Intervention + effect: QoL: *β* = 0.81 [−0.21, 1.84] *p* = .12 Composite *z*-score: *β* = 0.11 [0.01, 0.20] *p* = .03
**Lee et al.^49^**	RCT	398 (199), Community-dwelling older adults aged ≥65 with 3 chronic conditions (China)	73.2 (6.3), 60.1 ♀, Ethnicity unknown	12-month multidomain interventions included physical activities, cognitive training, and nutrition advice	IC: score consists of 5 domains, cognition, locomotion, vitality, psychology, and sensory domains; psychological and sensory scores multiplied by −1	Intervention + effect: *β* = 1.5 [0.5, 2.5] *p* = .005
** Chang et al.^54^**	CRT	567 (MIC 178, OC 201) from 30 centers, Community-dwelling individuals aged ≥50 (China)	MIC: 74.07 (8.91), 71.28 ♀, NM OC: 74.45 (8.39), 92.02 ♀, NM UC: 75.61 (8.53), 81.38 ♀, NM	3-year multicomponent integrated care (MIC): osteoporosis, sarcopenia, and polypharmacy pharmaceutical care, along with exercise training and nutritional support; or osteoporosis care (OC): osteoporosis treatment alone	IC: score consists of 5 domains, cognition, locomotion, vitality, psychology, and sensory domains, with each domain constructed into a binary scale (range: 0-6)	Intervention + effect: MIC: *β* = 0.3 [0.11] *p* = .01 OC: *β* = 0.0002 [0.10] *p* = 1
**Zhao et al.^51^**	CRT	81 (41) from 2 communities, community-dwelling older adults aged ≥60 with impairments in both locomotion and cognition (China)	Intervention: 75.95 (2.74), 75.6 ♀, Chinese Control: 75.33 (3.41), 77.5 ♀, Chinese	14-week nurse-led multidomain intervention including health education lectures, cognitive Training combined with multicomponent physical exercise, and personalized dietary plans	IC: score consists of 5 domains, cognition, locomotion, vitality, psychology, and sensory impairment	Intervention + effect: T0 baseline (*p* = .692) Intervention group: 6.56 (0.72) Control group: 6.63 (0.74) T1 post-intervention (*t* = 6.607, *p* < .001) Intervention group: 7.90 (1.02) Control group: 6.52 (0.68) Between-group effect: *F* = 38.948, *p* < .001 Time effect: *F* = 19.753, *p * < .001 Group × time interaction: *F* = 36.830, *p* < .001
**Interventions focused on physical exercise or activity**						
**Tarazona-Santabalbina et al.^45^**	CRT	100 (51) from 2 centers, Sedentary and frail individuals aged ≥70 (Spain)	Intervention: 79.7 (3.6), 56.9 ♀, NM Control: 80.3 (3.7), 51 ♀, NM	24-week multicomponent exercise program composed of endurance, strength, coordination, balance, and flexibility exercises	EuroQol quality-of-life (EQ-5D) Lawton and Brody scale	Intervention + effect Intervention Δ: EQ-5D: 7.4 (2.0) to 8.2 (1.6) Lawton and Brody scale: 6.7 (1.1) to 6.9 (0.9) Control Δ: EQ-5D: 7.7 (1.8) to 7.6 (1.3) Lawton and Brody scale: 6.8 (1.8) to 5.7 (2.0)
**Huang et al.^42^**	RCT	415 (AT 104, RT 102, AT+RT 104), Community-dwelling adults aged 65-85 with subjective memory concerns (Japan)	72.3 (4.6), 47 ♀, NM	26 weeks of aerobic and resistance training. AT: 60-min session consisted of a 10- to 15-minute step-in-place exercise and a 10- to 15-min walking workout, with a 5-min rest interval between training sets RT: 60-min session with resistance-band workouts and bodyweight exercises twice a week AT+RT: RT followed by AT	IC *z*-score: the mean *z*-score of 4 domains, cognition, locomotion, psychological, and vitality	+ effect for AT: Week 26: *β* = 0.19 [0.06, 0.32] p = .004; Week 52: *β* = 0.10 [0.03, 0.22] *p* = .12 + effect for RT: Week 26: *β* = 0.18 [0.07, 0.30] *p* = .002; Week 52: *β* = 0.13 [−0.01, 0.26] *p* = .06 No effect for AT+RT: Week 26: *β* = 0.05 [−0.07, 0.17] *p* = .40; Week 52: *β* = −0.01 [−0.13, 0.13] *p* = .99
**Sánchez-Sánchez et al.^44^**	RCT	188 (100), physically pre-frail/frail older individuals diagnosed with MCI/mild dementia (Spain)	84.06 (4.77), 72.29 ♀, NM	12-week Vivifrail multicomponent exercise program: home-based resistance, balance, flexibility, and walking exercise program tailored to an individual’s physical function	IC *z*-score: the mean *z*-score of 4 domains, cognition, locomotion, psychological, and vitality	Intervention + effect: *β* = 0.48 [0.24, 0.74] *p* < .001
**Huber et al.^56^**	RCT	88 (HG 42, FTG 46), inactive couples aged 50-60 (Italy)	HG: 58.57 (5.14), 50 ♀, NM FTG: 58.89 (5.67), 50 ♀, NM	7-day hiking or forest therapy: hiking group (HG): 3-4 hours daily guided, moderate hiking tours; forest therapy group (FTG): 3-4 hours daily nature therapy sessions, low physical activity, and connection therapy	Health-related QoL (SF-12) EuroQol (EQ-5D-5L index)	No significant effect for both SF-12 and EQ-5D-5L F1-LD-F1 model (*F* value) SF-12 T2 7-day 0.04 (1.00, ∞) *p* = .84 T4 180-day 0.43 (1.00, ∞) *p* = .51 EQ-5D-5L T2 7-day 0.01 (1.00, ∞) *p* = .92 T4 180-day 1.80 (1.00, ∞) *p* = .18
**Yıldırım Ayaz et al.^46^**	RCT	90 (AE 30, AE+RE 30), aged ≥65 with ability to perform moderate exercise and normal cognition levels (Turkey)	AE: 72.26 (6.41), 46.7 ♀, NM AE+RE: 74.43 (6.10), 43.3 ♀, NM Control: 75.33 (6.82), 20 ♀, NM	12-weeks individual or combined aerobic and resistance exercises (AE: aerobic exercises at 50-70% of maximum heart rate; RE: individualized low- and high-intensity resistance exercises)	IC *z*-score: *z*-score calculated from 5 domains, cognition, locomotion, vitality, psychology, and sensory domains	Individual and combined intervention + effect AE Δ: 0.09 (0.28) AE+RE Δ: 0.59 (0.32) Control Δ: −0.6 (0.30)
**Phillips et al.^43^**	RCT	49 (24), Insufficiently active females with metastatic breast cancer (USA)	54.8 (11.3), 100 ♀, 85.7 White	12-week Fit2ThriveMB to enhance physical activity, included weekly coaching calls, Fitbit, and a custom-built Social Cognitive Theory-guided Fit2ThriveMB app	Health-Related QoL (FACT-B): the Functional Assessment of Cancer Therapy-Breast (FACT-B) contained a 27-item scale	Intervention + effect Intervention Δ: 3.0 (2.3) Control Δ: 0.1 (2.1) *p* = .36
**Valenzuela et al.^52^**	Pooled analysis of 2 RCTs	570 (282), hospitalized older adults from 3 acute care for elders units aged ≥75 without complex medical needs (Spain)	Intervention: 87.4 (4.6), 50.0 ♀, NM Control: 87.3 (5.1), 45.5♀, NM	2.5-year multicomponent in-hospital exercise program including resistance, balance, and walking training	IC: score consists of 5 domains, cognition, locomotion, vitality, psychology, and sensory	Intervention + effect: *β* = 7.74 (6.45, 9.03) *p* < .001 Intervention Δ: 7.34 (5.70, 8.97) Control Δ: −0.41 (−2.00, 1.86)
**Caloric restriction**						
**Martin et al.^53^**	RCT	218 (143), healthy middle-aged adults with BMI 22.0 to 28.0 (USA)	Intervention: 38 (7.3), 69.2 ♀, 77.6 White Control: 37.9 (7.0), 70.7 ♀, 76 White	2-year 25% caloric restriction and recommendations of 30-min moderate physical activity per day, 5 days/week	QoL (SF-36) after 2 years	Intervention + effect: *β* = 6.45 (1.28) [3.93, 8.98] *p* < .001
**Supplementations**						
**Yi et al.^55^**	RCT	80 (60), healthy individuals aged 40-65 years with BMI between 18.5-35 kg/m^2^, (India)	Four groups 300 mg: 51.2 (7.0), 50 ♀, Indian 600 mg: 49.5 (6.7), 70 ♀, Indian 900 mg: 49.9 (6.3), 55 ♀, Indian Placebo: 46.5 (6.7), 60 ♀, Indian	60-day with daily oral NMN (3 doses: 300, 600, 900 mg) or placebo	QoL (SF-36)	All intervention dose + effect Placebo 129 ± 13 vs 300mg 137 ± 12 (*p* = .003); vs 600 mg 136 ± 12 (*p* < .001); vs 900mg 140 ± 11 (*p* < .001)

All studies are grouped by intervention type and then ordered according to publication time.

Abbreviations: AE, aerobic exercises; AT, aerobic training; BMI, body mass index; CRT, cluster randomized trial; NM, not mentioned in the study; QoL, quality of life; RCT, randomized controlled trial; RE, resistance exercise; RT, resistance training.

#### Randomized trial design

Eleven studies were randomized controlled trials, and 3 adopted a cluster randomized trial design.^45^^,^^51^^,^^54^ The 3 cluster randomized trials recruited participants from up to 30 health centers.^45^^,^^51^^,^^54^ Nine studies were two-arm, 2 were three-arm,^46^^,^^54^ 3 were four-arm.^42^^,^^48^^,^^55^ The 3 four-arm studies investigated either exercise or supplementation, or a combination of both.

#### Participants

The sample size of the included studies ranged from 49 to 1445. Participants were mainly older individuals, with more than half of the studies (*n* = 9) including adults with a mean age of over 70 years old. In contrast, one study recruited middle-aged participants with a mean age of 38. The majority of participants were community-dwelling, but some included individuals with specific chronic conditions, such as breast cancer^43^ or frailty.^45^ One study examining the effects of hiking on QoL specifically targeted physically inactive individuals.^56^

#### Interventions

Studies examined a range of interventions. The most common individual interventions were exercise (7 studies), which included aerobic, resistance, balance, or combined training; and nutrition (2 studies), which included caloric restriction and supplementation. However, the majority were multicomponent interventions that included various components such as physical activity, nutrition support, social interaction, and cognitive training. Overall, 12 of the 15 studies included an exercise component (8 as part of a multidomain intervention), 5 focused on nutrition (supplementation, caloric restriction, or advice), and 6 involved a cognitive training component. The duration of interventions also varied from 7 days to 3 years, and most were assessed immediately post-intervention, but some also longer-term.

#### Outcomes

Eight studies examined an IC score calculated from at least 4 IC domains (cognition, locomotion, vitality, psychological). Six studies examined QoL measures, but these differed across studies (2 used SF-36, 2 used EuroQoL, and 1 each for SF-12, SF-6D, FACT-B, PROMIS). Two studies used other multidimensional measures of healthspan, such as an active aging scale^47^ or a composite z-score (including assessment on physical activity, blood pressure, sleeping quality, use of medicine, depression, hemoglobin A_1c_, PROMIS, and smoking).^50^ Both of these measures captured multiple health domains and aligned with the healthspan concept, thus were eligible for inclusion in this review.

### Summary of outcome

Thirteen of the 15 studies reported that the interventions had significantly beneficial effects on indicators of healthspan. The results are summarized below by intervention type.

#### Exercise

From 7 studies with 1500 participants, exercise seemed to have a beneficial impact on IC and QoL (Table 2). Six out of 7 studies investigating various types of exercise reported a better outcome in the intervention group compared to the control group.^42–46^^,^^52^ However, only 1 of the 2 four-arm studies^42^^,^^46^ that examined resistance and aerobic exercise combined reported a positive effect on IC.^46^ Other studies assessed multicomponent exercise, including resistance, aerobic, balance, flexibility, and more.^43–45^^,^^52^ These studies all reported a small positive association between interventions and healthspan (Table 2). Sánchez-Sánchez et al. assessed the effect of exercise using IC,^44^ while Tarazona-Santabalbina et al. and Phillips et al. used QoL measurements.^43^^,^^45^ The study by Huber et al. compared hiking with forest therapy over 1 week and reported no significant effect on either of the QoL measures used (SF-12 and EuroQoL).^56^

**Table 2:** Summary of the effect of exercise alone or as part of a multicomponent intervention with others on healthspan.

Author	Intervention	Follow-up (weeks)	Measure of healthspan	Positive effect on healthspan
**Huang et al.^42^**	Aerobic and resistance training	52	IC	Yes
**Yıldırım Ayaz et al.^46^**	Aerobic and resistance training	12	IC	Yes
**Sánchez-Sánchez et al.^44^**	Multicomponent exercise	12	IC	Yes
**Tarazona-Santabalbina et al.^45^**	Multicomponent exercise	24	QoL (EuroQol and Lawton and Brody scale)	Yes
**Phillips et al.^43^**	Multicomponent exercise	12	QoL (FACT-B)	Yes
**Huber et al.^56^**	Hiking	1	QoL (SF-12 and EuroQol)	No
**Valenzuela et al.^52^**	Multicomponent in-hospital exercise	30	IC	Yes

#### Multicomponent intervention

Six studies with 2858 participants assessed the effectiveness of the intervention on indices of healthspan. All studies incorporated an exercise component; 5 out of 6 studies also included other aspects such as cognitive and stress training, and nutrition.^47–51^ One study specifically compared multicomponent integrated care (osteoporosis, sarcopenia, and polypharmacy pharmaceutical care with exercise training and nutritional support) and osteoporosis treatment only.^54^ Almost all studies reported a positive association between the intervention and healthspan, measured by IC, QoL, or a composite score derived by the authors (Table 3). Four studies investigated IC, and 3 reported that the intervention had a positive effect on IC compared with the control group,^49^^,^^51^^,^^54^ while 1 found no significant difference.^48^

**Table 3:** Summary of the effect of multidomain intervention on healthspan.

Author	Intervention	Follow-up (weeks)	Measure of healthspan	Positive effect on healthspan
**Giudici et al.^48^**	Multidomain intervention including exercise, cognitive training, and nutrition advice	52	IC	No
**Lee et al.^49^**	Multidomain intervention including exercise, cognitive training, and nutrition advice	52	IC	Yes
**Chang et al.^54^**	Multidomain intervention of integrated care	156	IC	Yes
**Yaffe et al.^50^**	Multidomain intervention including exercise, cognitive training, and nutrition advice	104	QoL, composite score derived by authors	Yes
**Chi et al.^47^**	Multidomain intervention including exercise, cognitive training	5	Active aging scale	Yes
**Zhao et al.^51^**	Multidomain, including cognitive training combined with physical exercise and health education lectures	14	IC	Yes

#### Supplementations

Two studies with a combined 1525 participants reported the effect of daily oral supplementation, β‑nicotinamide mononucleotide (NMN)^55^ and Omega-3 PUFA (ω-3),^48^ on indicators of healthspan. Various doses of NMN supplementation were all effective at improving QoL in the intervention groups compared to placebo over a 60-day period.^55^ Giudici et al. examined daily oral ω-3 supplementary for 12 months and reported no effect.^48^

#### Caloric restriction

One study examined 25% caloric restriction (CR) over 2 years and reported to result in significantly better SF-36 general health (effect estimate: 6.45, 95% CI: (3.93, 8.98) *p* < .001).^53^

### Risk of bias assessment

Most of the studies were ranked as having low to moderate risk of bias (Table S1). One study was rated as high risk of bias, mainly because the outcome measures were self-reported.^47^ The Lee et al. study^49^ had 4 out of 5 domains with moderate risk of bias, while another 5 studies had 1 or 2 domains classified as having moderate risk of bias.

## Discussion

This systematic review is so far the first to provide a comprehensive summary of the current evidence regarding the effects of various interventions on IC and QoL, as indicators of healthspan, in humans. It summarized the findings from 15 studies, including 4656 individuals aged predominantly 75 years or older (mean age range 38-84 years), with all but 1 study conducted in high-income or upper-middle-income countries. The most consistent evidence was that exercise alone or as part of a multidomain intervention, reported in 11 of 13 studies,^42–52^ was linked to better IC and QoL. Although the findings across these studies were statistically significant, the effect sizes were generally small, and therefore, the clinical significance of these differences is unclear. However, these findings were consistent with prior studies and together suggest the potential effectiveness of multidomain interventions incorporating exercise in prolonging functional indicators of healthspan. Other interventions, including caloric restriction and specific nutritional supplementation, also showed some effects, but conclusions could not be drawn due to the small number of studies and variability in both interventions and outcomes.^48^^,^^53^^,^^55^

Our finding that exercise interventions were found to have a potentially beneficial effect on IC and QoL aligns with recent cohort studies that have demonstrated a positive association between physical activity level and IC scores.^57–59^ A recent systematic review of 12 RCTs observed a positive effect of exercise on physical performance measured by the Short Physical Performance Battery among older adults.^60^ Further, our findings also echo two systematic reviews that demonstrated the effect of multidomain interventions, which included exercise, on domains of IC.^36^^,^^37^ Of the studies included in this review that found no effect of exercise, Huber et al. involved a slightly younger population (mean age: 59 years) and had a short 1-week hiking intervention.^56^ Giudici et al. used a multidomain intervention that included physical activity counseling, but reported improved IC across all groups, including the controls.^48^ Overall, our findings strengthen the existing evidence that exercise could be a potential way to enhance IC and QoL, and as a result, extend healthspan, when used alone or as part of a multidomain intervention.^28^^,^^57^^,^^61^

There are a few points to be noted from the findings of this review. Firstly, most studies implemented interventions after middle age, suggesting that such interventions were still effective even when initiated later in life. Moreover, the duration of the exercise and multidomain interventions lasted for at least 5 weeks to a maximum of 3 years for those studies reporting improved outcomes. The persistence of engagement in exercise and multidomain interventions may be critical to their effectiveness in promoting healthspan with respect to improved IC and QoL. Lastly, none of the included studies investigated cognitive training or nutrition alone, making it difficult to isolate their specific contributions within multidomain interventions. Therefore, the protective effect of multidomain interventions on IC and QoL may be predominantly due to the exercise component, and future research is needed to conclude the effect of other components in the multidomain intervention.

Although significant results were reported for CR and NMN supplementation in this review, there was only 1 study for each intervention. Diet is shown to be an important component that leads to good health by reducing the risk of chronic conditions.^62^ Prior evidence suggests that CR remains a well-established method for extending healthspan and promoting better health conditions.^63^^,^^64^ Similarly, Wickramasinghe et al. emphasized the importance of nutrition and a healthy diet on healthspan.^2^ Previous RCTs suggested NMN could improve physical function in humans.^65^ More RCTs would be needed to confirm the effect of dietary intervention, such as daily oral supplementation or CR, on improving an individual’s healthspan, assessed by holistic phenotypic outcomes.

Evidence from this review may be generalized to older Caucasians and Asians from high-income or upper-middle income countries, as the included studies were conducted in North America, Europe, and Asia. Findings cannot be generalized to other countries or younger adults. The studies included here recruited a range of community-dwelling individuals with various health conditions. However, due to the nature of exercise-related interventions, many studies excluded individuals with contraindications to physical activity.^42–44^ As a result, it was likely that participants in these trials had better baseline health conditions than their peers.

Although all included studies were randomized trials, potential recall bias may still arise from self-reported outcomes, and the inability to blind participants and observers to certain interventions (e.g., exercise, cognitive training).^66^ Previous research has highlighted that self-reported data tend to overestimate the effects due to reporting bias in RCTs.^67^ A few studies also identified the Hawthorne effect due to the awareness of participants in a trial, which could weaken the effect of interventions.

This review has several strengths. This systematic review was preregistered with PROSPERO (CRD420251015780)^38^ and conducted in accordance with the PRISMA guidelines.^39^ A comprehensive and reproducible search strategy was employed with defined inclusion and exclusion criteria, which included as many relevant articles as possible. All screening, data extraction, and RoB assessment were independently conducted by at least two reviewers. This reduced potential bias throughout the review process. RoB of the included studies was evaluated using a validated assessment tool. Besides, only RCTs were included, which are considered to be the gold standard for evaluating intervention effectiveness. These experimental study designs have the advantage of minimizing bias compared to observational studies.^68^ More importantly, the outcome measures defined in this review were multicomponent, mostly IC and QoL. In contrast, most other reviews only examined individual domains of IC rather than the integrated measure for healthspan.^36^^,^^37^ IC is a useful scale for evaluating one’s health as it provides a more comprehensive representation of preserved functioning level.^69^ Hence, a composite IC score would serve as a better indicator when assessing healthspan instead of single domains of IC (e.g., locomotion or cognitive domain alone). QoL, by contrast, offers a valuable representation of overall well-being from a person-centered perspective. Unlike IC, which primarily relies on objective tests, QoL provides an assessment of health from a distinct subjective viewpoint. Moreover, no restriction was made on the interventions being studied in this review. This maximized the possibility of including all relevant studies. Therefore, this review provided a comprehensive overview of current evidence on interventions extending healthspan, reflected by composite indicators such as IC and QoL.

Our review is also subject to a few limitations. There was no meta-analysis conducted due to the lack of consistency in both intervention and outcome measurements of the eligible studies, and no overall effect estimate was presented. All studies included were qualitatively described, with results presented individually. Moreover, although multiple studies employed IC as the outcome, variations in methods for assessing each IC domain could help explain some of the variation in the effect of IC across interventions and studies.^70^

## Conclusion

Exercise and multidomain interventions appeared to be effective at improving multidimensional indicators of healthspan, such as IC and QoL. There was limited existing evidence to conclude whether interventions such as CR and daily supplementation were beneficial for these multidimensional healthspan indicators, and further research in more diverse populations is also needed. As a new and emerging field, there are promising results from exercise and multidomain interventions, and multiple trials examining the effect of other interventions on IC are currently underway.^71–73^ Results from these ongoing IC-focused trials (e.g., a study examining the effect of combined exercise and cognitive intervention in older individuals)^72^ are expected within the next 2-3 years and will allow further quantitative synthesis. Identifying interventions that preserve and restore function at older age offers the potential to improve health status, reduce the risk of age-related diseases, and ultimately extend healthspan.

## Supplementary Material

glag133_Supplementary_Data

## Data Availability

No data were used in the research described in this article.
